# Supplementary material for: The association between body mass index and all-cause mortality in Japanese patients with incident hemodialysis
Source: PLoS One. 2022 Jun 24;17(6):e0269849. doi: 10.1371/journal.pone.0269849 (PMC9231701; doi:10.1371/journal.pone.0269849)
Supplement: Attachment — Submitted filename: Review BMI in HD Inagaki.docx [file pone.0269849.s002.docx]

I would like to review the manuscript submitted to *Plos One* by Koji Inagaki et al, Title; “The association between body mass index and all-cause mortality in Japanese patients with incident hemodialysis”.

In this paper, the authors reported that the association between BMI and all-cause deaths in incident dialysis patients in a single center retrospective cohort study. They showed that higher BMI measured 2 weeks after start of dialysis associated with longer survival. Thus, they concluded that “after the correction of volume overload, overweight was associated with lower mortality in Japanese patients with incident hemodialysis”.

Most of the previously papers related to BMI and prognosis in HD patients have been conducted at a time when dialysis conditions have stabilized. This is a study whether a condition at the time of initiation dialysis contribute to prognosis. If a good condition induced by a good pre-dialysis CKD management has an impact on subsequent life expectancy of patients, these results will be a strong message for readers including nephrologist of this journal.

But I have to state that the manuscript should not be acceptable in the present draft. The main problem is that there were two baselines to access mortality. And the discussion did not support to lead to the conclusion.

Major points

1. Which BMI did use to divide patients into three groups, at the first time HD session or 2 weeks after? Or, did the authors conduct survival analysis on two types of patient groups one from the other divided into three BMI categories? Did the authors do one survival analysis based on the data sheet divided into three groups by the first time BMI and another survival analysis based on the data sheet divided into three by the BMI after 2 weeks? If so, the authors should indicate not only the baseline data after 2 weeks of dialysis (Table1), but also the data after the start of dialysis. And The two survival analyzes should have different observation periods of 14 days. In particular, They should confirm whether the baseline data changes or not, since it was mentioned that the number of people in the three groups differs depending on when BMI was measured.
2. The authors stated that “the objective of the present study was to investigate the association between BMI and mortality in Japanese incident hemodialysis patients before and after the treatment of volume overload”. (line 66-68) and that “in our study, BMI decreased 2 weeks after HD initiation because the volume overload was corrected”. (line 246-247) . I agree that most patients probably improved their volume status. But, if they want to compare mortality between before and after the treatment of volume overload, they should evaluate and indicate data to support results that volume overload was corrected.

- Were all enrolled patients in condition with volume overload? They should indicate volume status at the first dialysis session.
- How many patients reached the adequate body weight after 2 weeks ultrafiltration treatment?.
- The author should show the source to decide “BMI after 2 weeks treatment”
- Also, the cardiothoracic ratio was relatively large in all groups in the data after 2 weeks of dialysis. They should show the data of cardiothoracic ratio decreased at the start of dialysis?
- Also, which body weight did they use to calculate BMI, before HD session or after HD session? We generally use body weight after HD session as dry weight = body weight without excess volume.

3. The authors used “BMI” and “overweight” as the same, but should use them separately. BMI is just a calculated number. High BMI include obesity, volume overload = overhydration, and overweight, and healthy with optimal weight, for instance high muscle mass. The unorganized and mixed terms makes readers confused.
4. I could not catch what was the aim of this study. The authors stated that “The objective of the present study was to investigate the association between BMI and mortality in Japanese incident hemodialysis patients before and after the treatment of volume overload”. But they discussed only the association between BMI and mortality while they referred the previous reports.

- One clinical implication of this study may be that more attention should be paid to HD patients with low BMI because they are very likely in malnutrition status as compatible many previous reports. Weight loss as a consequence of disease including CKD may induce the early mortality associated with a low BMI. Another implication may be that some HD patients with low BMI could have hidden protein energy waste or frail.
- And the authors should discuss why the association between low BMI after 2 weeks treatment and mortality disappeared.
- The authors should discuss the implication that association of higher BMI with lower death risk was more pronounced in elderly HD patients. Indeed, Japanese HD patients shows better survival that HD patients in Western countries. But it was not enough to explain the association of higher BMI with lower death risk.

5. I guess that the best survival prospects for incident dialysis patients may be those who need not to lose weight after initiation dialysis therapy because euvolemia, and those who are gain body weight because less uremia. Alternatively, I also guess that if patients are suffer from heart failure, the patients with better cardiac function can remove water more. So the larger lose weight may be better prognosis in patients with heart failure. In this study, I would like to see the data of changes in body weight during the two weeks and the association between these change and mortality.

Minor point

1. Six patients who died within three months were included as exclusion criteria. I think the main purpose of this study is to investigate the mortality of hemodialysis patients, so why did you exclude patients who died within 3 months? And was there any patients who died within 2 weeks?
2. The reference below (line 259-261) was not suitable for discussion about higher BMI with lower death risk. Add another proper one.

- Polinder-Bos et al. [22] reported that a lower BMI was associated with an increased 1-year mortality, but it was not associated with increased long-term mortality risk in elderly incident dialysis patients in the United States of America (USA).

3. The authors had better show cause of deaths.
