# Supplementary material for: The association between body mass index and all-cause mortality in Japanese patients with incident hemodialysis
Source: PLoS One. 2022 Jun 24;17(6):e0269849. doi: 10.1371/journal.pone.0269849 (PMC9231701; doi:10.1371/journal.pone.0269849)
Supplement: Attachment — Submitted filename: Response to reviewer.docx [file pone.0269849.s004.docx]

**Point-by-point responses to Reviewer’s comment**

We appreciate the time and efforts of the reviewer in reviewing this manuscript again. We have addressed all issues in the review report and believed that the revised version now meets the journal publication requirements.

**Comment & Response 3:** The authors should put the sentence “This is because the state of overhydration was controlled by 2 weeks after HD initiation in most patients [16] after the sentence “The data on body weight was obtained at the first HD session and 2 weeks after HD initiation.”

**Response:** We appreciate the reviewer’s valuable comments. Accordingly, we have changed the sentence as follows:

“The data on body weight was obtained at the first HD session and 2 weeks after HD initiation. This is because the state of overhydration was controlled by 2 weeks of HD initiation in most patients [16].” (page 6, lines 103–105).

**Comment & Response 6:** We still find the term “overweight” and “underweight” in the manuscript.

**Response:** We appreciate the reviewer’s careful observation and valuable comments on highlighting this issue. Accordingly, we have changed the terms “overweight” and “underweight” to “high BMI” and “low BMI,” respectively, in subsection “Mortality” of the “Results” section (page 15, lines 180–195).

**Comment & response 8:** The authors did not answer the question “why the association between low BMI after 2 weeks treatment and mortality disappeared”. The patients number of low BMI was increased after 2 weeks after HD initiation. Perhaps, I suppose most patients who lost weight could not eat enough by anorexia by uremia and in part dietary restriction pre and post dialysis initiation. But, this does not explain the question.

**Response**: We appreciate the reviewer’s valuable advice. We assumed that there was a large difference in lean body mass between low and normal BMI groups at HD initiation. However, there was a small difference in lean body mass between low and normal BMI groups at 2 weeks after HD initiation. Lean body mass is critical for skeletal muscle mass assessment [25]. Additionally, a low lean body mass is an independent risk factor for malnutrition [26]. Arase et al. [27] reported that a low lean body mass was associated with an increased risk for all-cause mortality in Japanese patients on maintenance HD. Out of 49 patients in the low BMI group at 2 weeks after HD initiation, 28 (57.1 %) belonged to the normal BMI group at the first HD session. We speculated that these patients would not have been excessively restricted nutritionally before HD initiation. As a result, even if these patients had undergone dialysis for 2 weeks to correct their fluid overload, they would have lost little muscle mass. Therefore, future studies need to investigate lean body mass in incident HD patients. We have added these sentences to our manuscript (page 22 line 279 to page 23 line 291) in the Discussion section.

**Comment & response 9:** Same as Q8, the authors did not answer the question “why association of higher BMI with lower death risk was more pronounced in elderly HD patients”

**Response:** We appreciate the reviewer’s valuable comment. We assumed that muscle and fat mass have a greater effect on mortality in elderly patients than in young patients. Thus, we revised the sentence as follows:

“In our study, the association of higher BMI with lower death risk was more pronounced in elderly HD patients. Similarly, Nagai et al. [28] reported that a high BMI was associated with an improved all-cause mortality in elderly patients undergoing HD, but not in middle-aged patients undergoing HD, in Japan. We speculated that muscle and fat mass have a greater effect on mortality in elderly patients than in young patients. Liu et al. reported that lean body mass had a greater effect on all-cause mortality in older adults than in younger adults [29]. Additionally, there was a monotonic positive association of fat mass with all-cause mortality in younger participants, but an approximate J-shaped trend in older participants [29]. In this study, high BMI groups in elderly patients had a higher serum Cr at 2 weeks after HD session than the other two groups. Additionally, only one of the 35 elderly patients in high BMI groups had a BMI >30 kg/m2. We speculate that older patients with high BMI had adequate muscle and fat mass. Therefore, adequate nutrition and exercise are important in elderly patients with pre-dialysis CKD” (page 23 line 292 to page 24 line 305) in the Discussion section.
